# Identification of Volatile Compounds and Terpene Synthase (*TPS*) Genes Reveals ZcTPS02 Involved in *β*-Ocimene Biosynthesis in *Zephyranthes candida*

**DOI:** 10.3390/genes15020185

**Published:** 2024-01-30

**Authors:** Guo Wei, Yang Xu, Mengmeng Xu, Xinwei Shi, Jianwen Wang, Liguo Feng

**Affiliations:** College of Horticulture and Landscape Architecture, Yangzhou University, Yangzhou 225009, China; gwei@yzu.edu.cn (G.W.); estherxu_2023@163.com (Y.X.); dx120230156@stu.yzu.edu.cn (M.X.); sxw19990119@163.com (X.S.); jwwang@yzu.edu.cn (J.W.)

**Keywords:** VOCs, terpene synthase, monoterpenes

## Abstract

*Zephyranthes candida* is a frequently cultivated ornamental plant containing several secondary metabolites, including alkaloids, flavonoids, and volatile organic compounds (VOCs). However, extensive research has been conducted only on non-VOCs found in the plant, whereas the production of VOCs and the molecular mechanisms underlying the biosynthesis of terpenes remain poorly understood. In this study, 17 volatile compounds were identified from *Z. candida* flowers using gas chromatography–mass spectrometry (GC-MS), with 16 of them being terpenoids. Transcriptome sequencing resulted in the identification of 17 terpene synthase (*TPS*) genes; two *TPS* genes, ZcTPS01 and ZcTPS02, had high expression levels. Biochemical characterization of two enzymes encoded by both genes revealed that *ZcTPS02* can catalyze geranyl diphosphate (GPP) into diverse products, among which is *β*-ocimene, which is the second most abundant compound found in *Z. candida* flowers. These results suggest that *ZcTPS02* plays a vital role in *β*-ocimene biosynthesis, providing valuable insights into terpene biosynthesis pathways in *Z. candida*. Furthermore, the expression of ZcTPS02 was upregulated after 2 h of methyl jasmonate (MeJA) treatment and downregulated after 4 h of the same treatment.

## 1. Introduction

*Z. candida*, commonly known as the white rain lily, is a perennial bulbous plant belonging to the Amaryllidaceae family and originates from South America. Its hardiness, adaptability to various environmental conditions, and extended blooming period make it an exceptional ornamental plant that is frequently utilized in urban landscaping worldwide. In addition to its decorative value, *Z. candida* contains various secondary metabolites, including alkaloids [1], flavonoids, and volatile organic compounds (VOCs), which potentially contribute to its medicinal properties. Traditional medicine practices have employed it for diverse therapeutic purposes because of diuretic, emmenagogue and antirheumatic properties [2]. Research into *Z. candida* has focused on cultivation management [3], pest control, and chemical compounds [4]. Despite the extensive knowledge of the traditional uses of *Z. candida*, its VOC profile and the intricate molecular mechanisms underlying the biosynthesis of these VOC metabolites, particularly the terpenoids, remain poorly understood.

Terpenoids belong to the most abundant and structurally diverse class of secondary metabolites found in plants, with over 80,000 compounds reported [5,6]. Except for a small number of terpenes involved in plant primary metabolism (e.g., gibberellin and sterols), most terpenes serve various functions, such as attracting beneficial allies or repelling natural enemies [7,8,9,10]. While the cytoplasmic mevalonate (MVA) pathway produces precursors for sesquiterpene and triterpene biosynthesis, the plastidic 2-C-methyl-d-erythritol 4-phosphate (MEP) pathway generates precursors that are responsible for monoterpene and diterpene biosynthesis [11,12]. Terpene synthases (TPSs) are pivotal enzymes in terpene biosynthesis, catalyzing the conversion of geranyl diphosphate (GPP, C_10_), farnesyl diphosphate (FPP, C_15_), geranylgeranyl diphosphate (GGPP, C_20_), and geranylfarnesyl diphosphate (GFPP, C_25_) into monoterpenes, sesquiterpenes, diterpenes, and sesterterpenes, respectively [13,14,15,16]. While the number of *TPS* genes in a plant’s family varies among species, they share a common evolutionary origin [17,18,19]. Based on phylogenetic analysis, the TPS family can be divided into seven subfamilies (TPS-a to TPS-g) which possess distinct functions and taxonomic distributions. In Angiosperms, proteins encoded by TPS-a produce sesquiterpenes, whereas those encoded by TPS-b and TPS-g produce monoterpenes [18]. TPS enzymes are classified into two types: type I and type II. Type I TPS enzymes have an aspartate-rich DDxx(D,E) motif in their C-terminal domains, which is essential for binding a metal cofactor (Mg^2+^ or Mn^2+^) [5]. Type II terpene synthases have a DxDD motif near the N-terminus. The motif’s second aspartate is crucial for the protonation-initiated cyclization of GGPP. Advancements in genome and transcriptome sequencing have facilitated the identification of additional functions of TPS enzymes in plants. For instance, the gene clusters responsible for sesterterpene production in *Arabidopsis thaliana* have an impact on the plant’s root microbiota [20]. In rice, several *TPS* genes are insect- or fungus-induced, and are expressed for the formation of volatile terpenes [21]. In maize, most *TPS* genes are responsible for producing volatile terpenoids induced by herbivory [22,23]. Despite the rich secondary metabolites produced by *Z. candida*, little is known about the profile of VOCs and the molecular mechanisms underlying terpene biosynthesis in this plant. This study has two primary objectives: to comprehensively determine the volatile compounds present in *Z. candida* flowers, and to elucidate the terpene biosynthesis in *Z. candida*. These volatile compounds, particularly terpenoids, play a crucial role in the plant’s ecological interactions with its environment and greatly contribute to distinctive floral fragrances that have captivated human fascination for centuries. Using advanced chemical analysis techniques, including Solid Phase Microextraction Gas Chromatography–Mass Spectrometry (SPME-GC-MS), the complex combinations of terpenoids and other volatile compounds produced by *Z. candida* flowers can be revealed. This study’s secondary aim was to explore the genetic basis of monoterpene production in *Z. candida* by identifying the *TPS* genes responsible for this process. Through the extensive selection of potential genes, a particular *TPS* gene, named *β*-ocimene synthase, is responsible for the production of *β*-ocimene, which is a crucial monoterpene present in *Z. candida* blooms.

## 2. Results

### 2.1. Determination of Volatile Compounds in Z. candida Flowers

**Figure 1:**
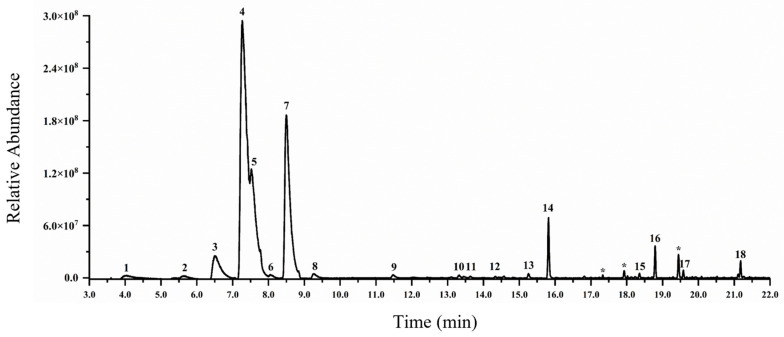
GC-MS analysis of volatiles from *Z. candida* flowers, showing the gas chromatographs of volatile compounds emitted from *Z. candida* flowers. The numbers (ordered by retention time) represent the peaks of volatiles. 1, *α*-thujene; 2, *β*-thujene; 3, *β*-myrcene; 4, D-limonene; 5, *β*-terpinyl acetate; 6, *α*-pinene; 7, *β*-ocimene; 8, (+)-4-carenel; 9, allo-ocimene; 10, limonene oxide; 11, (+)-(E)-limonene oxide; 12, myroxide; 13, (E)-9-octadecene; 14, linalool; 15, humulene; 16, *α*-terpineol; 17, *α*-farnesene; 18, (E)-*β*-ocimene. *, impurities.

The volatile compounds of *Z. candida*, particularly terpenoids, were collected using fused silica fibers coated with divinylbenzene/carboxen/polydimethylsiloxane (DVB/CAR/PDMS) and analyzed by gas chromatography–mass spectrometry (GC-MS). Volatile compounds were tentatively identified by the spectral matching of analytical spectra with reference mass spectra in the National Institute of Standards and Technology 08 database (Figure 1). A total of 17 volatile compounds were tentatively identified in *Z. candida* flowers, 16 of which were terpenoids. Among these, 10 were monoterpenes, including *α*-thujene, *β*-thujene, *β*-myrcene, D-limonene, *α*-pinene, *β*-ocimene, (+)-4-carenel, allo-ocimene, linalool, and *α*-terpineol. Four monoterpene derivatives were identified, including cyclohexanol, *β*-terpinyl acetate, limonene oxide, (+)-(E)-limonene oxide, and myroxide. Two sesquiterpenes, humulene and *α*-farnesene, were identified. The major monoterpenes observed in the sample were D-limonene, *β*-ocimene, and linalool. The only non-terpene compound was (*E*)-9-octadecene (peak 13), which belongs to the alkenes.

### 2.2. Identification of Terpene Synthase Genes in Z. candida

To investigate terpene biosynthesis in *Z. candida*, transcriptome sequencing was performed; a total of 42,679,098 raw reads were generated and subsequently processed using Fastp for the removal of adapters, low-quality sequences, and unknown sequences. A total of 42,416,162 clean reads were obtained, which were assembled into 186,527 unigenes and 297,622 transcripts with an N50 of 750 and an average length of 571 bp. After redundancy elimination, TransDecoder software (version 5.5.0) predicted 42,322 open reading frames (ORFs) from the transcripts. The Q20 (%) and Q30 (%) quality scores were 97.92 and 93.77, respectively.

**Figure 2:**
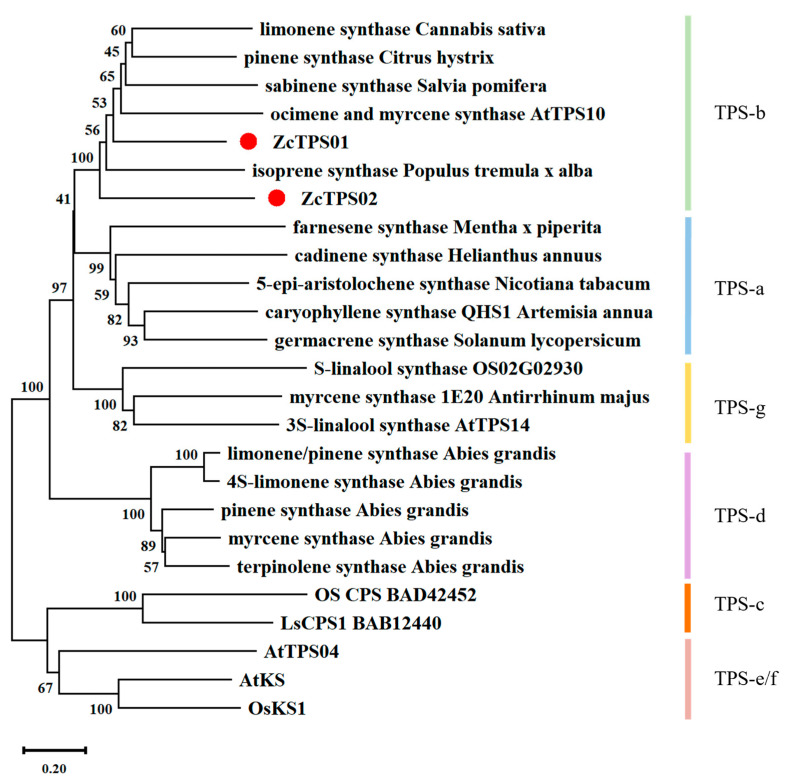
Phylogenetic tree analysis. The phylogenetic tree was constructed using the 1000 repeated neighborhood connection method. Bootstrap values exceeding 40% are indicated for the respective nodes. The scale quantifies the evolutionary distance in substitutions per site. Color classifications represent the TPS-a to TPS-g subfamilies. The TPS-b subfamily is represented by green, TPS-a by blue, TPS-g by orange, TPS-d by purple, TPS-c by red, and TPS-e/f by pink. The red dots indicate the two sequences studied in this paper. At, *Arabidopsis thaliana*; Os, *Oryza sativa*.

**Figure 3:**
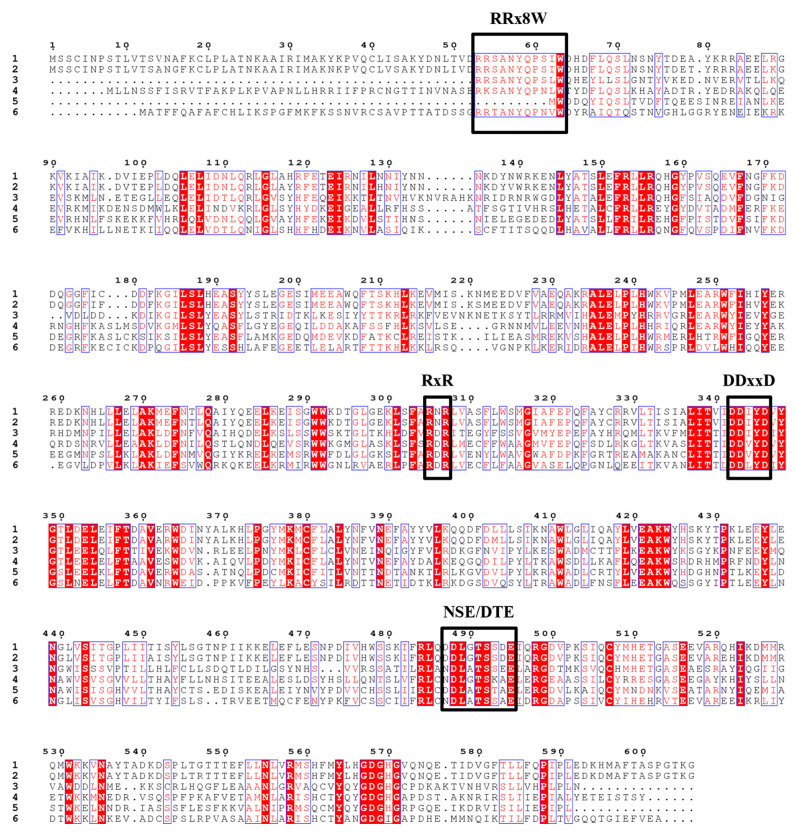
Sequence alignment analysis. Alignment of *Z. candida* TPS amino acid sequences with functionally characterized TPSs from other species. The numbers in the figure represent the amino acid sequences. 1, (+)-limonene synthase 1 (*Citrus limon*) (accession no. AAM53944); 2, (+)-limonene synthase 2 (*Citrus limon*) (accession no. AAM53946); 3, E-*β*-ocimene synthase, partial (*Arabidopsis thaliana*) (accession no. AAN65379); 4, *β*-ocimene synthase (*Phaseolus lunatus*) (accession no. ABY65110); 5, ZcTPS01; 6, ZcTPS02. The sequences were aligned by Genomenet and analyzed with ESPript 3.0. The red background shading represents 100% identity and light red represents 70% identity. The blue frame shows the similarity across groups. The terpene synthases motifs RRx_8_W, RxR, DDxxD, and NSE/DTE are indicated by the black box.

To identify terpene synthase genes in *Z. candida*, the BLASTP algorithm was used with Arabidopsis *TPS* genes as queries. A total of 17 unigenes were identified as TPSs, and five the unigenes appeared to be in full length. Two genes, TRINITY_DN524_c0_g1_i4.p1 and TRINITY_DN1469_c0_g1_i2.p1, which were designated as *ZcTPS01* and *ZcTPS02*, respectively, were selected as candidate genes because of their high expression levels. The fragments per kilobase of the exon model per million mapped fragments (FPKM) of ZcTPS01 and ZcTPS02 were 2141.42 and 1214.48, respectively. The FPKMs of the other three genes (TRINITY_DN16452_c2_g1_i4_p1, TRINITY_DN12218_c0_g1_i2_p1, and TRINI-TY_DN64768_c0_g2_i7_p1) were quite low (<10). Phylogenetic analysis showed that the two TPSs belong to the TPS-b subfamily (Figure 2), indicating that these two TPSs may be responsible for monoterpene formation in *Z. candida*. The length of ZcTPS01 was 1719 bp with 573 amino acids encoded, while ZcTPS02 had a total length of 1767 bp and encoded 589 amino acids. Sequence alignment analysis showed that ZcTPS02 features the RRx_8_W motif whereas ZcTPS01 does not (Figure 3). The proteins encoded by ZcTPS01 and ZcTPS02 contained the aspartate-rich region DDxxD and the NSE/DTE motif, which coordinate a trinuclear Mg^2+^ cluster that binds to the diphosphate prior to ionization [24,25]. Both ZcTPS01 and ZcTPS02 have another conserved sequence, known as the RxR motif, which is responsible for binding the diphosphate group after substrate ionization [26].

### 2.3. Biochemical Characterization of Enzymes Encoded by ZcTPS

**Figure 4:**
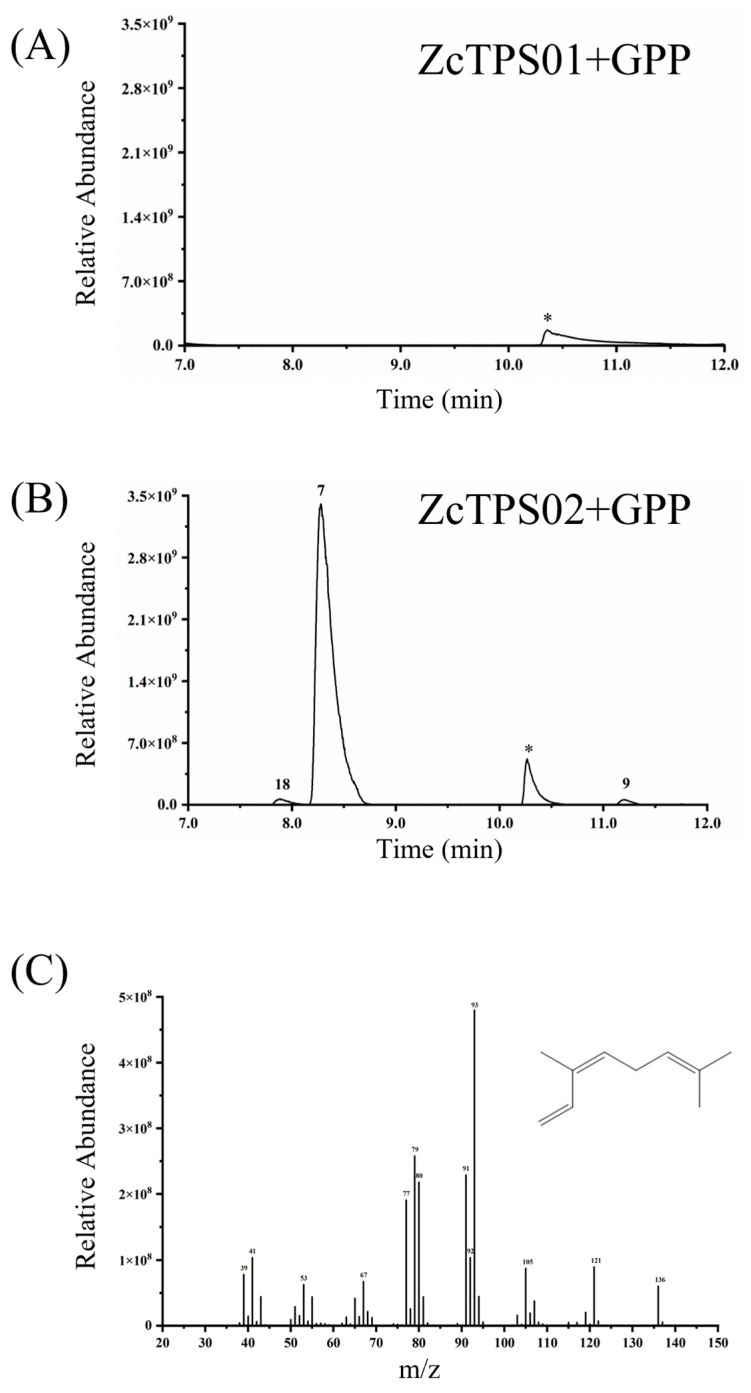
Biochemical characterization of enzymes encoded by ZcTPS. In vitro enzymatic assays of ZcTPSs were performed and the resulting enzyme products were collected by SPME and subsequently analyzed through GC-MS. (**A**) Chromatogram of terpenes generated by the ZcTPS01 protein purified from BL21(DE3) pLysS cells in a reaction with GPP. (**B**) Chromatogram of terpenes generated by the ZcTPS02 protein from BL21(DE3) pLysS cells in a reaction with GPP. (**C**) Chemical structure and mass spectra of *β*-ocimene. 7, *β*-ocimene; 9, allo-ocimene; 18, (E)-*β*-ocimene. *, impurities.

To investigate the molecular mechanism underlying the biosynthesis of monoterpenes in *Z. candida* flowers, the full-length coding regions of ZcTPS01 and ZcTPS02 were cloned and expressed in *Escherichia coli*. The resulting recombinant ZcTPS proteins were tested with GPP. While ZcTPS01 did not show any activity toward GPP, ZcTPS02 was able to catalyze GPP into several products, with *β*-ocimene as the major product, which is consistent with its presence in the flower of *Z. candida*. The other products were allo-ocimene and (*E*)-*β*-ocimene (Figure 4). Despite attempting to determine the function of ZcTPS01 utilizing various methods, such as the use of the MBP-tagged vector and truncated proteins, challenges in obtaining soluble protein were encountered. The ZcTPS02 protein was tested with FPP as the substrate and did not produce any products with FPP. This result suggests that ZcTPS02 is specific to monoterpene formation.

### 2.4. Subcellular Localization of ZcTPS02

**Figure 5:**
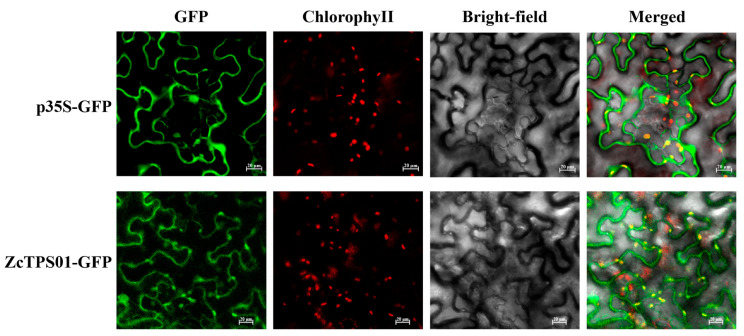
Subcellular localization of ZcTPS02. After infiltration for 36 h with GV3101, pictures were obtained using a confocal laser microscope. The upper panel shows pCAMBIA 1300-35S-sGFP transformed tobacco leaf cells and the lower panel shows ZcTPS02-GFP transformed cells. The GFP emission signal in the left panel is green, the middle is chlorophyll red fluorescence and bright-field, and the two signals on the right overlap. Bars, 20 µm.

GPP is a crucial precursor in the biosynthesis of monoterpenes, which are generally believed to be synthesized via the plastidMEP pathway [27]. In this study, Cell-PLoc 2.0 and WoLF PSORT II were employed to predict the subcellular localization of ZcTPS02. ZcTPS02 was predicted to be located mainly in chloroplasts. To further investigate the subcellular localization of ZcTPS02, ZcTPS02 was fused to green fluorescent protein (GFP) protein and expressed in *Nicotina. benthamiana* leaves. The computational prediction tools and experimental analyses consistently indicated that ZcTPS02 is primarily located in the plastid, implying that ZcTPS02 is responsible for the production of monoterpenes in *Z. candida* (Figure 5).

### 2.5. Expression Analysis of ZcTPS02 Gene under Methyl Jasmonate (MeJA) Treatment

**Figure 6:**
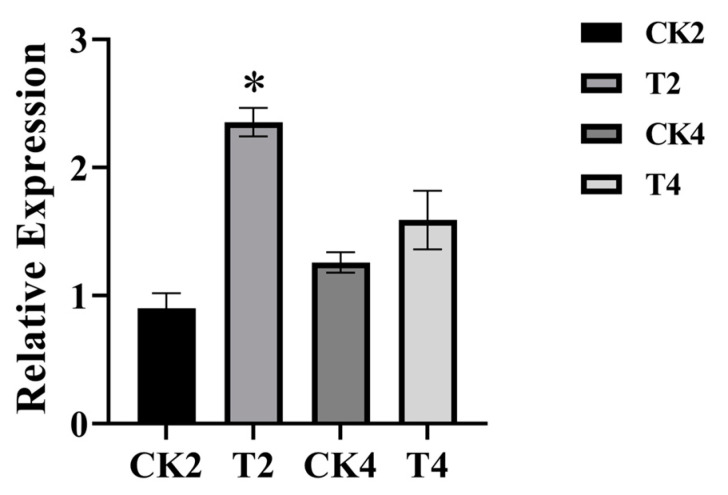
Relative expression of ZcTPS02 after MeJA treatment. The expression of ZcTPS02 after MeJA treatment. Error bars represent the SD of three independent experiments. CK2 and CK4 were treated with 0.1% ethanol for 2 and 4 h. T2 and T4 were treated with MeJA for 2 and 4 h. The * indicates a significant difference (*p* < 0.05).

Terpenoid synthase genes play a crucial role in biological stress. Further experimentation was conducted to investigate whether ZcTPS02 functions similarly. To simulate exogenous biological stress, the petals were treated with methyl jasmonate (MeJA) and quantitative reverse transcription-polymerase chain reaction (qRT-PCR) analysis was performed. The results indicated that there was an approximate 2.5-fold increase in *ZcTPS02* gene expression level within 2 h after MeJA treatment, while the gene expression level was unchanged after 4 h of treatment (Figure 6).

## 3. Materials and Methods

### 3.1. Materials and Reagents

*Z. candida* was collected from the Wenhui campus at YangZhou University (32.391° N, 119.419° E), Yangzhou, China. The samples were promptly frozen in liquid nitrogen and subsequently maintained at −80 °C for RNA extraction. GPP, FPP, and MeJA were purchased from Sigma-Aldrich (Merck, Darmstadt, Germany).

### 3.2. Determination of Volatile Compounds in Z. candida

To investigate the volatile compounds in *Z. candida*, the whole flowers were collected during the flowering period. The detached flowers were placed in a beaker. Subsequently, the beaker was tightly sealed with aluminum foil. The SPME needle was carefully inserted through the aluminum foil and fixed in place. The SPME fiber was extended into the headspace of the culture to initiate the collection of volatile compounds and subsequently analyzed using GC-MS (Clarus SQ8T, PerkinElmer, Waltham, MA, USA) [28]. At the start of experiment, the temperature was increased to 50 °C and maintained there for 1 min. The temperature was increased to 120 °C at a rate of 5 °C per minute, then to 200 °C at a rate of 8 °C per minute. Finally, the temperature was raised to 250 °C at a rate of 12 °C per minute and held for 7 min. The MS conditions were an emission current of 200 μA, ionization energy of 70 eV, and mass scan range of 29 to 600 amu.

### 3.3. Transcriptome Analysis

Total RNA extraction was carried out using poly-T oligo-attached magnetic beads (Invitrogen, Waltham, MA, USA), and the subsequent construction of the cDNA library was performed according to the protocol of the TruSeq stranded mRNA library prep kit (Illumina, San Diego, CA, USA). After filtering the raw reads by Fastp [29], the Q20, Q30, and GC percentages of the clean reads were calculated and evaluated. For de novo reference transcriptome assembly, the clean reads obtained from sequencing and filtering were pooled and assembled using Trinity software (v2.0.13) [30]. The longest transcripts were extracted by cd-hit and redundant sequences were removed. All the ORFs of the transcripts were finally predicted by TransDecoder. Trinity was utilized to assess transcript expression levels through RNA-Seq by expectation maximization.

### 3.4. In Silico Analysis of ZcTPSs and Phylogenetic Analysis

Sequences, namely (+)-limonene synthase 1 (*Citrus limon*) (AAM53944), (+)-limonene synthase 2 (*Citrus limon*) AAM53946), E-*β*-ocimene synthase, partial (*Arabidopsis thaliana*) (AAN65379), *β*-ocimene synthase (*Phaseolus lunatus*) (ABY65110), and ZcTPSs, were aligned using Genomenet (https://www.genome.jp/. 3 March 2023) and analyzed with ESPript 3.0 (https://espript.ibcp.fr. 3 March 2023). Typical TPS sequences obtained from a previous study [31] were utilized for the evolutionary tree analysis performed by MEGA 11, and 1000 bootstrap value repetitions were used. The GenBank accession numbers for phylogenetic tree analysis were as follows: limonene synthase *Cannabis sativa*, ABI21837; pinene synthase *Citrus hystrix*, ADX01381; sabinene synthase *Salvia pomifera*, ABH07678; ocimene and myrcene synthase AtTPS10, Q9ZUH4; isoprene synthase *Populus tremula x alba*, CAC35696; farnesene synthase *Mentha x piperita*, AAB95209; cadinene synthase *Helianthus annuus*, ACA33926; 5-epi-aristolochene synthase *Nicotiana tabacum*, AFJ04408; caryophyllene synthase QHS1 *Artemisia annua*, AAL79181; germacrene synthase *Solanum lycopersicum*, AEM05858; S-linalool synthase Os02g02930, NP_001396182; myrcene synthase 1E20 *Antirrhinum majus*, AAO41727; 3S-linalool synthase AtTPS14, Q84UV0; limonene/pinene synthase *Abies grandis*, Q9M7C9; 4S-limonene synthase *Abies grandis*, AAB70907; pinene synthase *Abies grandis*, AAB71085; myrcene synthase *Abies grandis*, AAB71084; terpinolene synthase *Abies grandis*, AAF61454; OS CPS BAD42452, BAD42452; LsCPS1 BAB12440, BAB12440; AtTPS04, Q93YV0; AtKS, Q9SAK2; OsKS1, NP_001389353.

### 3.5. Gene Cloning and Vector Construction

Total RNA was extracted from *Z. candida* flowers with a TaKaRa MiniBEST plant RNA extraction kit (TaKaRa, Beijing, China). Reverse transcription was performed using the PrimeScript II 1st Strand cDNA Synthesis Kit (TaKaRa, Beijing, China) according to the manufacturer’s instructions. Primers (Supplemental Table S1) for ZcTPSs were designed using the NEB Tm Calculator (https://tmcalculator.neb.com/. 5 March 2023), and the resulting cDNA was cloned into the pEASY-Blunt E1 expression vector (TransGen Biotech, Beijing, China) and transformed into DH5α chemically competent cells. The resulting plasmids were sequenced by Sangon Biotech. Sequence-correct plasmid was extracted using a TIANprep Mini Plasmid Kit (TIANGEN, Beijing, China). For homologous recombination, the Vazyme ClonExpress Ultra One Step Cloning Kit (Vazyme, Nanjing, China) was utilized.

### 3.6. Crude Bacterial Lysate Extraction and GC-MS Analysis

The plasmid was extracted from bacteria and transformed into BL21(DE3) pLysS according to the protocol. To initiate growth, 1 mL fresh bacteria was added with the plasmid to 50 mL of liquid medium resistant to carbenicillin and chloramphenicol in a sterilized flask. The flask was then incubated at 37 °C and 200 rpm for 3 h. After incubation, 0.3 mM isopropyl *β*-D-thiogalactopyranoside (IPTG) was added to the bacterial culture, which was incubated at 16 °C and 150 rpm for 12 h. After centrifugation at 5000 rpm for 5 min, the supernatant was discarded. The *E. coli* cells were disrupted using a JY96-IIN probe sonicator (SCIENTZ) for forty cycles of 9 s each in chilled extraction buffer. After centrifugation, the crude bacterial lysate was desalted using spinOUT columns (Sangon, Shanghai, China). Enzymatic assays were conducted as previously described [32]. In summary, a reaction mixture of 100 µL was prepared consisting of 25 mM MOPS (pH 7.0), 10 mM MgCl_2_, 2 mM DTT, 40 μM GPP and/or (*E*,*E*)-FPP, and 50 µL of crude bacterial lysates. The mixture was then incubated at 30 °C for 2 h. Volatile compounds were collected using SPME and subjected to GC-MS analysis [33,34].

### 3.7. Subcellular Localization of ZcTPS

Cell-PLoc 2.0 (http://www.csbio.sjtu.edu.cn/. 15 March 2023) and WoLF PSORT II (https://wolfpsort.hgc.jp/. 15 March 2023) were used to predict the subcellular localization of ZcTPS02.

The pCAMBIA 1300-35S-sGFP vector was digested with the restriction enzymes Sac I and Xba I. The *ZcTPS02* gene was then inserted into the pCAMBIA 1300-35S-sGFP vector. After sequencing to confirm successful insertion, the vector was transferred to *Agrobacterium* GV3101 and cultured on plates containing kanamycin and rifampicin for 2–3 days. The *Agrobacterium* containing ZcTPS02-GFP and the *Agrobacterium* containing the *P19* gene were incubated at 28 °C and 200 rpm until they reached OD_600_ = 0.5–0.6, respectively. After centrifugation at 5000 rpm for 10 min, both were resuspended in infiltration buffer containing 0.5 mM MES (pH 5.6), 0.2 mM acetosyringone (AS), and 1 mM MgCl_2_, adjusted to OD_600_ = 1.0, mixed in equal volumes, and left at room temperature for 2–3 h. The epidermis of the tobacco leaves was infiltrated and the plants were grown under normal lighting for 36–48 h after an 8-h dark period. The GFP fluorescence signal was observed using a Zeiss LSM 880 laser confocal microscope [35].

### 3.8. MeJA Treatment

First, 0.1% Methyl Jasmonate (MeJA) was evenly sprayed onto the petals of *Z. candida* with consistent growth; 0.1% ethanol was used as the control treatment. Samples were collected at 2 and 4 h after treatment. RNA was extracted from the MeJA-treated and control samples using an RNA extraction kit and then reverse transcribed to cDNA using the HiScript II Q Select RT SuperMix for qPCR kit (Vazyme, Nanjing, China). Gene expression was quantified through the use of the SYBR Green qPCR Master Mix kit (Vazyme, Nanjing, China) and subsequent data analysis was accomplished through a relative quantitative approach and graphing utilizing GraphPad Prism 9 (https://www.graphpad.com/. 1 November 2023).

## 4. Discussion

The primary objective of this study was to investigate the biosynthesis of terpenes in *Z. candida* by identifying and characterizing the *TPS* genes and their encoded enzymes. SPME-GC-MS analysis showed that 16 out of the 17 identified volatile compounds were terpenoids, with D-limonene, *β*-ocimene, and linalool being the most abundant monoterpenes. To date, more than 1700 VOCs from 90 different plant families have been categorized. These plant VOCs are divided into three main classes based on their biosynthetic origin: terpenoids, phenylpropanoids/benzenoids, and alcohols/aldehydes [36]. The dominance of terpenoid metabolic pathways in *Z. candida* flowers is indicated by the high proportion of terpenoids (16/17). Their potential significance in the ecological context of *Z. candida* and their potential value for use in the fragrance and flavor industry are indicated by this study. In flowering plants, terpenoids are widely present in the volatile metabolites of plants. For example, during the bloom period, over 20 volatile terpenes are released from *Clematis florida* [37]. The wild species of the *Freesia* genus displays a variety of terpenes [38]. Notably, the identification of four monoterpene derivatives in *Z. candida* suggests that monoterpene skeletons can be modified by other enzymes, including cytochrome P450 oxygenases (CYPs) and acyltransferases, and form a wider range of volatile compounds [39]. In addition to volatile compounds, biosynthetic pathways for nonvolatile specialized metabolites in plants should be further elucidated [40].

Through transcriptome sequencing, 17 unigenes were identified as potential TPSs. The *TPS* gene family has been extensively studied in various plant species, with reported repertoires ranging from tens to hundreds of *TPS* genes [18,35,41]. For the gene expression analysis of the transcriptome, ZcTPS01 and ZcTPS02 were selected for further investigation. Sequence alignment results showed the presence of several typical monoterpene synthase motifs. Notably, the RR_X8_W motif, located at the protein’s N-terminus, is a hallmark of the N-terminal domain and has been demonstrated to undergo isomerization in the initial step from geranyl diphosphate to linalyl diphosphate [42,43,44]. The RR_X8_W motif is fully conserved and found in ZcTPS02, but it is absent in ZcTPS01; this finding may explain the unsuccessful characterization of the biochemical function of ZcTPS01. Phylogenetic analysis result showed that ZcTPS01 and ZcTPS02 belong to the TPS-b subfamily, which is distinct from the TPS-g group that includes (E)-*β*-ocimene synthase (encoded by Arabidopsis AtTPS14) and (E)-*β*-ocimene synthase (ama0a23) [45]. After examining the TPS-g subfamily of *Z. candida*, only one TPS-g gene (TRINITY_DN1687_c0_g1. P1) is present in this clade. However, this gene seems to be a partial sequence with a remarkably low expression level. It is worth noting that (E)-*β*-ocimene synthases have emerged multiple times in separate lineages and have undergone convergent evolution at the molecular scale [46]. The enzymatic assay result showed that ZcTPS02 produces *β*-ocimene, which has been detected in the flowers of *Z. candida*, suggesting that ZcTPS02 may produce this product in vivo. Despite numerous attempts, ZcTPS01 has not yet yielded any enzymatic products. Considering that information about terpene metabolism in *Z. candida* is limited, future studies may uncover enzymatic activity and enhance our understanding of the complex molecular networks involved in plant terpene biosynthesis. For example, ten monoterpenes were detected in this study, indicating a high rate of GPP formation in the flowers of *Z. candida*. The molecular mechanism of GPP formation, specifically the action of geranyl diphosphate synthase (GPPS), requires further investigation. Most GPPS has been found in plastids for GPP formation, and cytosolic RcG/FPPS1 produces GPP in the MVA pathway for monoterpene biosynthesis in rose flowers [47].

(E)-*β*-ocimene is a compound emitted by plants in response to external stimuli. In *Arabidopsis thaliana*, the expression level of AtTPS03 was upregulated in leaves after mechanical wounding and treatment with jasmonic acid [48]. When tea plants were exposed to tea geometrid attack, *β*-ocimene synthase (CsBOS1) displayed a pattern of varying levels, with low concentrations during the day and high concentrations at night [49]. Additionally, transgenic torenia plants enriched with (E)-*β*-ocimene showed enhanced ability to attract the predatory mite *Phytoseiulus persimilis* [50]. These findings have been observed in various plant systems, highlighting the importance of ocimene in mediating plant responses to environmental cues and interactions within the surrounding ecosystem. Therefore, further investigation is necessary to explore the ecological significance of ocimene in *Z. candida*. Understanding the ecological significance of ocimene in *Z. candida* may reveal new aspects of its interaction with the environment and its role in attracting beneficial organisms or repelling potential threats. Another interesting question is whether ocimene is involved in inter-organ aerial transport [51]. This study demonstrated a 2.5-fold increase in ZcTPS02 expression after 2 h of MeJA treatment, suggesting an important role in the response of *Z. candida* to biological stress.

## 5. Conclusions

In conclusion, the volatile organic compounds in *Z. candida* flowers were successfully identified, with most of these compounds found to be terpenoids. Transcriptome sequencing was conducted to further investigate the molecular mechanism underlying the terpene biosynthesis, resulting in the identification of 17 *TPS* genes. Sequence alignment showed that several typical TPS motifs are present in the ZcTPS01 and ZcTPS02. In the biochemical assay, while ZcTPS01 did not show catalytic activity, ZcTPS02 was demonstrated to be involved in β-ocimene biosynthesis in *Z. candida*. Furthermore, ZcTPS02 was significantly upregulated by MeJA treatment at 2 h. This study provides insights into how terpenes are produced in *Z. candida* and establishes a foundation for further investigations into the biochemical and molecular mechanisms that underlie terpene production in various plant species. This study can have significant implications for multiple industries, particularly the fragrance and flavor sectors, as terpenes are highly valued in these industries due to their diverse and unique properties.

## Data Availability

Raw RNA-seq data were deposited in the SRA database (PRJNA1019494).

## Supplementary Materials

The following supporting information can be downloaded at: https://www.mdpi.com/article/10.3390/genes15020185/s1, Table S1: Primers used in this study.
